# Use of statins and risks of ovarian, uterine, and cervical diseases: a cohort study in the UK Biobank

**DOI:** 10.1007/s00228-024-03656-y

**Published:** 2024-02-28

**Authors:** Xue-Feng Jiao, Hailong Li, Linan Zeng, Huazhen Yang, Yao Hu, Yuanyuan Qu, Wenwen Chen, Yajing Sun, Wei Zhang, Xiaoxi Zeng, Lingli Zhang

**Affiliations:** 1 Department of Pharmacy, West China Second University Hospital, Sichuan University, Chengdu, Sichuan China; 2 Evidence-Based Pharmacy Center, West China Second University Hospital, Sichuan University, Chengdu, Sichuan China; 3 NMPA Key Laboratory for Technical Research On Drug Products In Vitro and In Vivo Correlation, Chengdu, Sichuan China; 4 Key Laboratory of Birth Defects and Related Diseases of Women and Children, Sichuan University, Ministry of Education, Chengdu, Sichuan China; 5 West China Biomedical Big Data Center, West China Hospital, Sichuan University, Chengdu, Sichuan China; 6 Medical Big Data Center, Sichuan University, Chengdu, Sichuan China; 7 Division of Nephrology, Kidney Research Institute, West China Hospital, Sichuan University, Chengdu, Sichuan China; 8 Chinese Evidence-Based Medicine Center, West China Hospital, Sichuan University, Chengdu, Sichuan China

**Keywords:** Statins, Cohort study, Risk, Cervical cancer, Polycystic ovarian syndrome

## Abstract

**Purpose:**

To examine the associations between use of statins and risks of various ovarian, uterine, and cervical diseases, including ovarian cancer, endometrial cancer, cervical cancer, ovarian cyst, polycystic ovarian syndrome, endometriosis, endometrial hyperplasia, endometrial polyp, and cervical polyp.

**Methods:**

We conducted a cohort study among female participants in the UK Biobank. Information on the use of statins was collected through verbal interview. Outcome information was obtained by linking to national cancer registry data and hospital inpatient data. We used Cox proportional hazards regression to examine the associations.

**Results:**

A total of 180,855 female participants (18,403 statin users and 162,452 non-users) were included. Use of statins was significantly associated with increased risks of cervical cancer (adjusted hazard ratio (HR), 1.55; 95% confidence interval (95% CI), 1.05–2.30) and polycystic ovarian syndrome (adjusted HR, 4.39; 95% CI, 1.68–11.49). However, we observed no significant association between use of statins and risk of ovarian cancer, endometrial cancer, ovarian cyst, endometriosis, endometrial hyperplasia, endometrial polyp, or cervical polyp.

**Conclusion:**

Our findings suggest that use of statins is associated with increased risks of cervical cancer and polycystic ovarian syndrome, but is not associated with increased or decreased risk of ovarian cancer, endometrial cancer, ovarian cyst, endometriosis, endometrial polyp, or cervical polyp.

**Supplementary Information:**

The online version contains supplementary material available at 10.1007/s00228-024-03656-y.

## Introduction

Statins, as inhibitors of 3-hydroxy-3-methyl-glutaryl coenzyme A (HMG-CoA) reductase, function by impeding the biosynthesis of cholesterol through the inhibition of the conversion of HMG-CoA to mevalonate. Consequently, they are primarily used in the treatment of hypercholesterolemia and for the secondary prevention of coronary artery diseases. Statins are among the most widely prescribed drugs worldwide [1, 2]. For example, in the United States, an estimated 38.7 million persons, about 12% of the population, were taking a statin [3].

In addition to their lipid-lowering effect, statins exhibit other pleiotropic effects. For example, some experimental studies of human cell lines and animal models suggest that statins may have beneficial effects in the prevention and treatment of several ovarian and uterine diseases, such as ovarian cancer, endometrial cancer, polycystic ovary syndrome, and endometriosis [4, 5]. However, clinical studies regarding this issue are scarce and have yielded inconsistent results [5].

On the other hand, the pleiotropic effects of statins are not always considered beneficial in previous studies. For example, some other experimental studies of human cell lines and animal models have reported the toxic effects of statins on the ovary and uterus. These toxic effects include antiproliferative and pro-apoptotic effects on ovarian and endometrial cells, inhibition of ovarian steroidogenesis, morphological and histological changes in the ovary, antiangiogenic effects, and reduced fertility [6, 7]. Moreover, in our prior pharmacovigilance study, by disproportionality analyses using the FDA Adverse Event Reporting System (FAERS) database, we found that use of statins might be associated with increased risks of ovarian cancer, endometrial cancer, cervical cancer, ovarian cyst, polycystic ovarian syndrome, endometriosis, endometrial hyperplasia, endometrial polyp, and cervical polyp [8]. However, the results of disproportionality analyses could only demonstrate statistical associations and not causations and should be verified by further cohort studies [9].

The UK Biobank is a large-scale database containing extensive sociodemographic, lifestyle, and clinical data on half a million participants. Leveraging this database, we conducted a cohort study to comprehensively examine the associations between use of statins and risks of ovarian cancer, endometrial cancer, cervical cancer, ovarian cyst, polycystic ovarian syndrome, endometriosis, endometrial hyperplasia, endometrial polyp, and cervical polyp.

## Methods

### Data source

The UK Biobank comprises 502,507 volunteer participants aged 37–73 from England, Scotland, and Wales who were recruited between 2006 and 2010. Details of the design and survey methods for UK Biobank have been described in previous studies [10, 11]. At baseline assessment visit and repeat assessment visits, participants completed a touchscreen questionnaire and a verbal interview, which collected information on sociodemographic characteristics, lifestyle, medical history, medication history, and reproductive factors. Repeat assessment visits were conducted every 2–3 years during the follow-up period, at which participants underwent a repetition of the baseline assessment visit. Thus, repeat assessment visits could enrich, confirm, and calibrate the data collected at baseline assessment visit. Moreover, touchscreen questionnaire validation was performed in two ways. First, some questions (especially medical questions) in the touchscreen questionnaire would be asked again and confirmed in the subsequent verbal interview. Second, the touchscreen questionnaire incorporated a number of logic checks on the data that were entered, such as checking for contradictory answers and impossible or improbable numeric values [12].

In addition, the collected data were linked to hospital inpatient data, national cancer registry data, and national death registry data, which enabled long-term follow-up of participants and their health-related outcomes. Hospital inpatient data on participants in England, Scotland, and Wales were received from their respective databases: the Hospital Episode Statistics for England (HES), the Scottish Morbidity Record (SMR), and the Patient Episode Database for Wales (PEDW) [9]. National cancer registry data and national death registry data were acquired from the National Health Service (NHS) Digital (for participants in England or Wales) and the NHS Central Register (for participants in Scotland) [13].

### Study design and population

We conducted a cohort study of female participants in the UK Biobank. We excluded females who had a history of cancer (except for non-melanoma skin cancer) [14], ovarian cyst, polycystic ovarian syndrome, endometriosis, endometrial hyperplasia, endometrial polyp, cervical polyp, ovariectomy, hysterectomy, or cervicectomy at baseline, or who had withdrawn from the UK Biobank. The required information was collected through touchscreen questionnaire/verbal interview and linkage to hospital inpatient data and national cancer registry data. Details of the variable name, data field, and data coding in the UK Biobank are given in Supplemental Table 1.

### Exposure

Information on the use of statins was self-reported and collected through verbal interview. If the participant indicated in the touchscreen that they were taking cholesterol-lowering drugs, then the interviewer was prompted to record the name of the drug. Use of statins was defined as continuous use of statins for months or years. It did not include the use of statins for a few days or a week, or prescribed statins that were not taken [15]. Based on treatment with a statin or not, the participants were divided into statin users and non-users. The statins recorded in the UK Biobank included simvastatin, atorvastatin, rosuvastatin, and pravastatin (Supplemental Table 2). Specific data on usage, dosage, and duration were not recorded.

### Outcome

The outcomes were first diagnoses of ovarian cancer, endometrial cancer, cervical cancer, ovarian cyst, polycystic ovarian syndrome, endometriosis, endometrial hyperplasia, endometrial polyp, and cervical polyp during the follow-up period. Cases of incident ovarian cancer, endometrial cancer, and cervical cancer were ascertained by linking to national cancer registry data and hospital inpatient data, and incident ovarian cyst, polycystic ovarian syndrome, endometriosis, endometrial hyperplasia, endometrial polyp, and cervical polyp were ascertained by linking to hospital inpatient data. We also obtained the first diagnosis date from national cancer registry data and hospital inpatient data. The corresponding variable name, data field, and data coding in the UK Biobank are presented in Supplemental Table 3.

### Follow-up time

When assessing cancer outcomes, female participants were followed from baseline visit until the first diagnosis of the outcome, the diagnosis of other cancer (except for non-melanoma skin cancer), death, or the last linkage date with national cancer registry data and hospital inpatient data (31 December 2016 for national cancer registry data, 31 March 2017 for HES, 31 October 2016 for SMR, or 29 February 2016 for PEDW), whichever came first [13, 16]. In addition, when assessing non-cancer outcomes, female participants were followed from baseline visit until the first diagnosis of the outcome, death, or the last linkage date with hospital inpatient data (31 March 2017 for HES, 31 October 2016 for SMR or 29 February 2016 for PEDW), whichever came first [13, 16]. The required information was obtained by linking to national cancer registry data, hospital inpatient data, and national death registry data.

### Covariates

The covariates included age, race (white or others), Townsend deprivation index (quintiles), smoking status (never, past, or current), alcohol use (daily or almost daily, three or four times a week, once or twice a week, one to three times a month, special occasions only, or never), vigorous physical activity (low, moderate, or high), number of childbirth, number of abortion, comorbidities at baseline (hyperlipidemia, ischemic heart disease, ischemic cerebrovascular disease, hypertension, diabetes, obesity, or pelvic inflammatory disease), and oral contraceptive. These covariates were factors known to be correlated with risks of all outcomes according to previous literatures, or indications for use of statins [4]. Moreover, for each outcome, we included extra related covariates which were correlated solely with risk of this outcome according to previous literatures (Supplemental Table 4). All these covariates were collected through touchscreen questionnaire/verbal interview and linkage to hospital inpatient data. Details of the variable name, data field and data coding in the UK Biobank are given in Supplemental Table 5. The Townsend deprivation index was widely used as a measure of socioeconomic deprivation, with higher scores indicating greater deprivation [17]. The number of childbirth was derived from the number of live births and stillbirths. In addition, the number of abortion was derived from the number of spontaneous miscarriages and pregnancy terminations. Furthermore, obesity was defined as body mass index (BMI) ≥ 30. Missing data were coded as a missing indicator category for categorical variables and with mean values for continuous variables.

### Statistical analysis

#### Baseline analysis

Comparisons were made between satin users and non-users for the following variables at baseline: age, race, Townsend deprivation index, smoking status, alcohol use, vigorous physical activity, number of childbirth, number of abortion, comorbidities (hyperlipidemia, ischemic heart disease, ischemic cerebrovascular disease, hypertension, diabetes, obesity, and pelvic inflammatory disease), and oral contraceptive. Continuous variables were presented as mean (standard deviation (SD)) and analyzed by using the Student’s *t*-test or median (interquartile range (IQR)) and by Wilcoxon rank-sum test, as appropriate. Categorical variables were presented as counts and percentages and evaluated by chi-square test, Fisher’s exact test, or rank-sum test as appropriate.

#### Main analysis

We used Cox proportional hazards regression to analyze the associations between use of statins and risks of ovarian, uterine, and cervical diseases, with results expressed as hazard ratios (HRs) and 95% confidence intervals (95% CI). Time since baseline visit was used as the underlying timescale. We developed a multivariable model with adjustment for age, race, Townsend deprivation index, smoking status, alcohol use, vigorous physical activity, number of childbirth, number of abortion, any comorbidity at baseline (hyperlipidemia, ischemic heart disease, ischemic cerebrovascular disease, hypertension, diabetes, obesity, or pelvic inflammatory disease), and oral contraceptive. Moreover, for better control of some outcome-specific confounders, we included extra related covariates in the Cox proportional hazards model for each outcome (Supplemental Table 4). Furthermore, the analyses of ovarian cyst, polycystic ovarian syndrome, and endometriosis were restricted to the premenopausal female cohort because these diseases are less likely to develop after menopause.

#### Subgroup analysis

We used Schoenfeld residuals to test the proportional hazards assumption and found that the assumption was violated for age. Thus, we performed subgroup analysis stratified by age to assess if change in result was noteworthy. We performed subgroup analysis stratified by the median age (age (≤ 56 or > 56 years) for ovarian cancer, endometrial cancer, cervical cancer, endometrial hyperplasia, endometrial polyp, and cervical polyp; age (≤ 46 or > 46 years) for ovarian cyst, polycystic ovarian syndrome, and endometriosis). In addition, to assess the potential modification effects by statin type, we performed subgroup analysis among different statins.

#### Sensitivity analysis

We conducted several sensitivity analyses to confirm the robustness of the results. First, to minimize the potential for reverse causality, we performed a sensitivity analysis by excluding the first year of follow-up (for all individuals). Second, to minimize indication bias, we performed a sensitivity analysis by restricting the study population to females with hyperlipidemia, ischemic heart disease, ischemic cerebrovascular disease, hypertension, diabetes, or obesity (all these diseases are indications for use of statins or common comorbidities in statin users). Third, as the average age of menopause in UK women is 51 years [18], we performed a sensitivity analysis by censoring the follow-up at age 51 for the outcomes of ovarian cyst, polycystic ovarian syndrome, and endometriosis.

All data analyses were conducted using R version 3.6.3. Statistical significance was set at *P* < 0.05 using two-sided tests. However, as the threshold of *P* < 0.05 is conventional and arbitrary, it does not convey any meaningful evidence of clinical significance or the size of the effect. Thus, we comprehensively examined the precise *P* values, the estimates of the effect sizes, and the confidence intervals, to interpret the statistical analyses and evaluate the clinical significances [19].

## Results

**Fig. 1:**
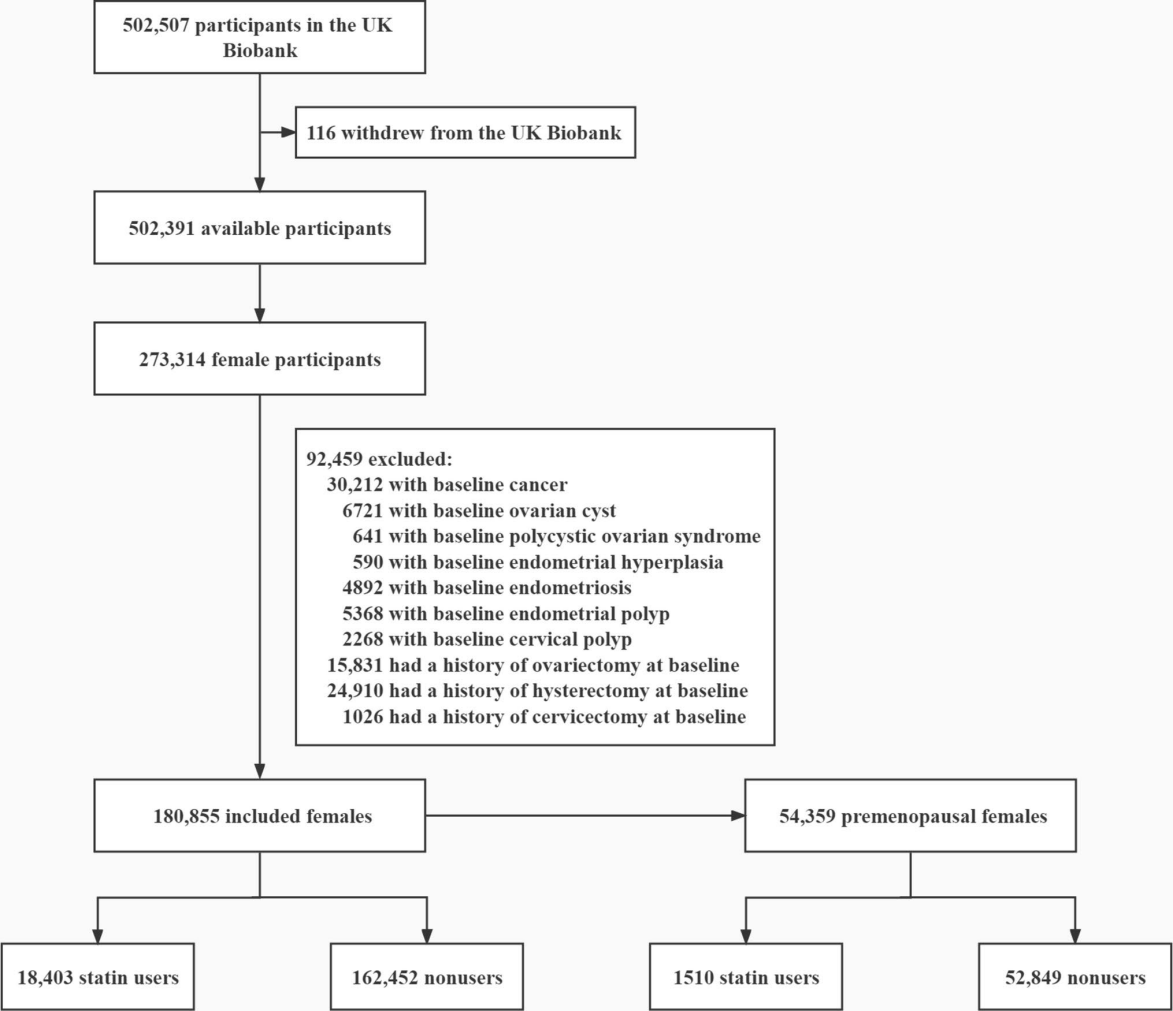
Flow chart of study population

Our study identified 273,314 female participants in the UK Biobank. Among these, 92,459 were excluded because of having a history of cancer (except for non-melanoma skin cancer), ovarian cyst, polycystic ovarian syndrome, endometriosis, endometrial hyperplasia, endometrial polyp, cervical polyp, ovariectomy, hysterectomy, or cervicectomy at baseline. In total, 180,855 female participants were included in analysis (18,403 statin users and 162,452 non-users) (Fig. 1). The median age of the included participants was 56 years (IQR, 49–62) at baseline. Among them, 54,359 participants (1510 statin users and 52,849 non-users) were premenopausal females, and their median age was 46 years (IQR, 43–49) at baseline. Table 1 describes the baseline characteristics of participants according to use of statins. Compared with non-users, statin users were more likely to be older, socioeconomically deprived, and smokers. They also had higher number of childbirth and more comorbidities. Moreover, statin users were less likely to be white and physical active, yet had fewer alcohol consumption, lower number of abortion, and less use of oral contraceptives. In addition, when we restricted the study population to premenopausal females, there were no significant differences between statin users and non-users in smoking status, number of childbirth, or number of abortion, while the characteristics of other covariates were similar to the whole study population (Supplemental Table 6).

**Table 1:** Baseline characteristics of participants by use of statins

Characteristics	Non-users (*n* = 162,452)	Statin users (*n* = 18,403)	*P* value
Age, years, median (IQR)	55 (48–61)	62 (57–66)	< 0.001
Race			< 0.001
White	152,657 (94.0)	17,118 (93.0)	
Others	8972 (5.5)	1202 (6.5)	
Missing	823 (0.5)	83 (0.5)	
Townsend deprivation index (quintiles)			< 0.001
1 (least deprived)	33,061 (20.4)	3104 (16.9)	
2	32,757 (20.2)	3333 (18.1)	
3	32,536 (20.0)	3588 (19.5)	
4	32,352 (19.9)	3773 (20.5)	
5 (most deprived)	31,537 (19.4)	4589 (24.9)	
Missing	209 (0.1)	16 (0.1)	
Smoking status			< 0.001
Never	99,523 (61.3)	10,158 (55.2)	
Past	48,284 (29.7)	6384 (34.7)	
Current	13,802 (8.5)	1752 (9.5)	
Missing	843 (0.5)	109 (0.6)	
Alcohol use			< 0.001
Daily or almost daily	26,988 (16.6)	2657 (14.4)	
Three or four times a week	35,612 (21.9)	3055 (16.6)	
Once or twice a week	42,661 (26.3)	4206 (22.9)	
One to three times a month	20,931 (12.9)	2360 (12.8)	
Special occasions only	22,209 (13.7)	3539 (19.2)	
Never	13,571 (8.4)	2528 (13.7)	
Missing	480 (0.3)	58 (0.3)	
Vigorous physical activity			< 0.001
Low	22,797 (14.0)	2911 (15.8)	
Moderate	55,190 (34.0)	5849 (31.8)	
High	49,685 (30.6)	4655 (25.3)	
Missing	34,780 (21.4)	4988 (27.1)	
Number of childbirth, median (IQR)	2 (1–2)	2 (1–3)	< 0.001
Number of abortion, median (IQR)	0 (0–1)	0 (0–1)	< 0.001
Comorbidities			
Hyperlipidemia	3944 (2.4)	12,767 (69.4)	< 0.001
Ischemic heart disease	1827 (1.1)	2680 (14.6)	< 0.001
Ischemic cerebrovascular disease	860 (0.5)	1153 (6.3)	< 0.001
Hypertension	29,333 (18.1)	11,024 (59.9)	< 0.001
Diabetes	2408 (1.5)	3719 (20.2)	< 0.001
Obesity	32,385 (19.9)	6992 (38.0)	< 0.001
Pelvic inflammatory disease	1759 (1.1)	161 (0.9)	0.010
Oral contraceptive			< 0.001
Yes	134,183 (82.6)	13,259 (72.0)	
No	27,398 (16.9)	5031 (27.3)	
Missing	871 (0.5)	113 (0.6)	

*IQR* interquartile range. Data are *n* (%) unless otherwise indicated

Table 2 shows the results of main analysis. During a median follow-up of 8–9 years, the numbers of female participants with a first diagnosis of ovarian cancer, endometrial cancer, cervical cancer, ovarian cyst, polycystic ovarian syndrome, endometriosis, endometrial hyperplasia, endometrial polyp, and cervical polyp were 599, 849, 363, 601, 32, 528, 397, 3166, and 814, respectively. The crude incidence per 1000 person-years among non-users and statin users was 0.41 compared to 0.56 for ovarian cancer, 0.55 compared to 1.09 for endometrial cancer, 0.26 compared to 0.24 for cervical cancer, 1.37 compared to 1.39 for ovarian cyst, 0.06 compared to 0.49 for polycystic ovarian syndrome, 1.20 compared to 1.23 for endometriosis, 0.26 compared to 0.39 for endometrial hyperplasia, 2.15 compared to 2.58 for endometrial polyp, and 0.56 compared to 0.52 for cervical polyp. After adjustment for the covariates, use of statins was significantly associated with increased risks of cervical cancer (adjusted HR, 1.55; 95% CI, 1.05–2.30) and polycystic ovarian syndrome (adjusted HR, 4.39; 95% CI, 1.68–11.49). However, we observed no significant association between use of statins and risk of ovarian cancer (adjusted HR, 0.94; 95% CI, 0.73–1.22), endometrial cancer (adjusted HR, 1.06; 95% CI, 0.88–1.28), ovarian cyst (adjusted HR, 0.92; 95% CI, 0.56–1.52), endometriosis (adjusted HR, 0.84; 95% CI, 0.49–1.42), endometrial hyperplasia (adjusted HR, 1.04; 95% CI, 0.77–1.40), endometrial polyp (adjusted HR, 0.99; 95% CI, 0.88–1.11), or cervical polyp (adjusted HR, 0.99; 95% CI, 0.76–1.28).

**Table 2:** The associations between use of statins and risks of ovarian cancer, endometrial cancer, cervical cancer, ovarian cyst, polycystic ovarian syndrome, endometriosis, endometrial hyperplasia, endometrial polyp, and cervical polyp

Outcome	Non-users			Statin users			Adjusted*	
	No. of participants	No. of outcome	Incidence per 1000 person-years	No. of participants	No. of outcome	Incidence per 1000 person-years	HR (95% CI)	*P*
Ovarian cancer	162,452	519	0.41	18,403	80	0.56	0.94 (0.73–1.22)	0.665
Endometrial cancer	162,452	693	0.55	18,403	156	1.09	1.06 (0.88–1.28)	0.541
Cervical cancer	162,452	329	0.26	18,403	34	0.24	1.55 (1.05–2.30)	0.028
Ovarian cyst	52,849	584	1.37	1510	17	1.39	0.92 (0.56–1.52)	0.758
Polycystic ovarian syndrome	52,849	26	0.06	1510	6	0.49	4.39 (1.68–11.49)	0.003
Endometriosis	52,849	513	1.20	1510	15	1.23	0.84 (0.49–1.42)	0.503
Endometrial hyperplasia	162,452	340	0.26	18,403	57	0.39	1.04 (0.77–1.40)	0.819
Endometrial polyp	162,452	2790	2.15	18,403	376	2.58	0.99 (0.88–1.11)	0.883
Cervical polyp	162,452	738	0.56	18,403	76	0.52	0.99 (0.76–1.28)	0.926

*HR* hazard ratio, *CI* confidence interval

*Adjusted for age, race, Townsend deprivation index, smoking status, alcohol use, vigorous physical activity, number of childbirth, number of abortion, any comorbidity at baseline (hyperlipidemia, ischemic heart disease, ischemic cerebrovascular disease, hypertension, diabetes, obesity, or pelvic inflammatory disease), oral contraceptive, and extra outcome-specific covariates. Moreover, the analyses of ovarian cyst, polycystic ovarian syndrome, and endometriosis were restricted to the premenopausal female cohort

**Fig. 2:**
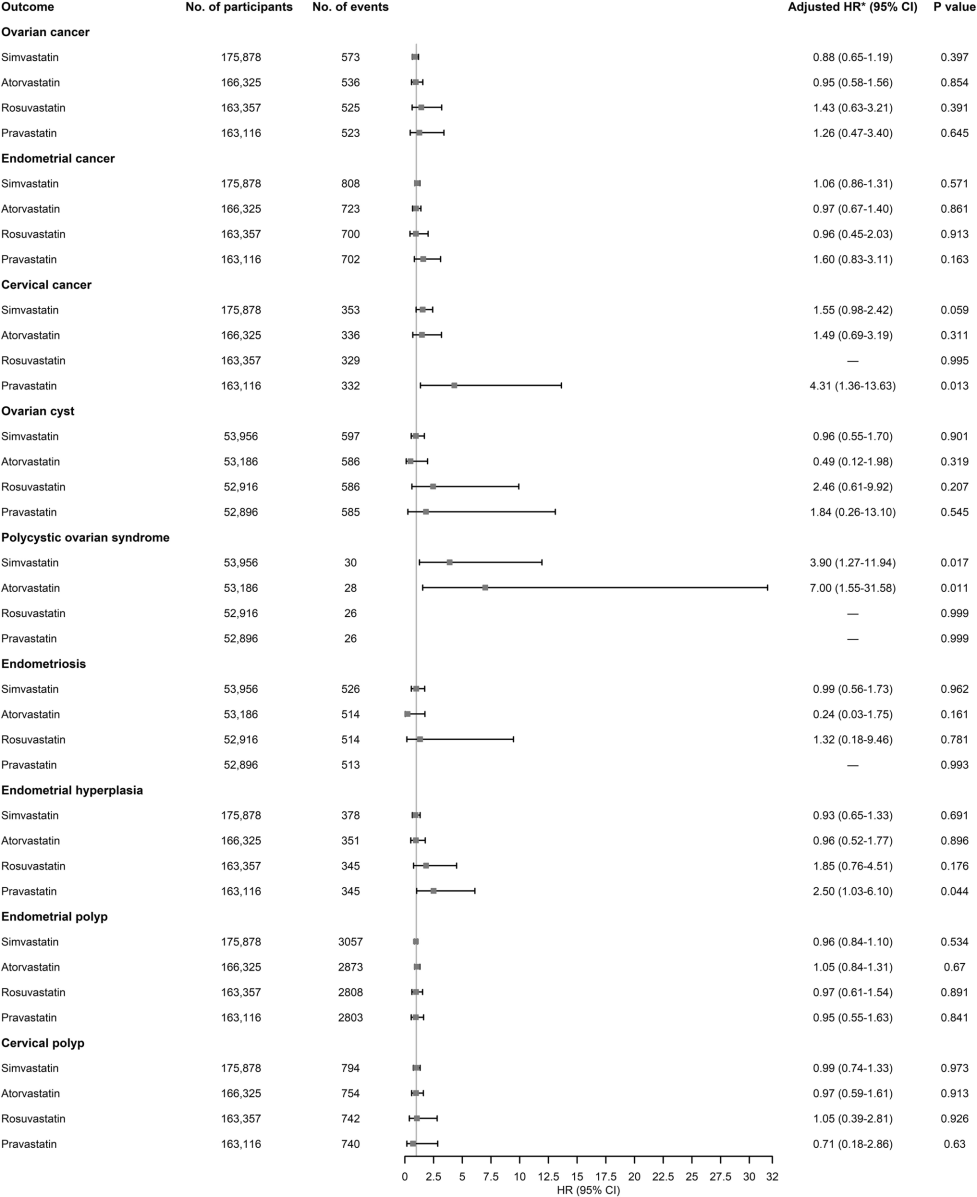
The associations between use of statins and risks of ovarian cancer, endometrial cancer, cervical cancer, ovarian cyst, polycystic ovarian syndrome, endometriosis, endometrial hyperplasia, endometrial polyp, and cervical polyp stratified by statin type. HR hazard ratio, CI confidence interval; —, the sample size was too small to enable statistical analysis. *Adjusted for age, race, Townsend deprivation index, smoking status, alcohol use, vigorous physical activity, number of childbirth, number of abortion, any comorbidity at baseline (hyperlipidemia, ischemic heart disease, ischemic cerebrovascular disease, hypertension, diabetes, obesity, or pelvic inflammatory disease), oral contraceptive, and extra outcome-specific covariates. Moreover, the analyses of ovarian cyst, polycystic ovarian syndrome, and endometriosis were restricted to the premenopausal female cohort

Figure 2 shows stratified analyses by statin type. The numbers of simvastatin, atorvastatin, rosuvastatin, and pravastatin users in the subgroups were 13,426, 3873, 905, and 664, respectively. When we restricted the study population to premenopausal females, the numbers of simvastatin, atorvastatin, rosuvastatin, and pravastatin users in the subgroups were 1107, 337, 67, and 47, respectively. For cervical cancer, use of pravastatin was significantly associated with increased risk of cervical cancer (adjusted HR, 4.31; 95% CI, 1.36–13.63), use of simvastatin was borderline associated with increased risk of cervical cancer (adjusted HR, 1.55; 95% CI, 0.98–2.42), whereas use of atorvastatin was not significantly associated with risk of cervical cancer. For polycystic ovarian syndrome, uses of simvastatin (adjusted HR, 3.90; 95% CI, 1.27–11.94) and atorvastatin (adjusted HR, 7.00; 95% CI, 1.55–31.58) were all significantly associated with increased risk of polycystic ovarian syndrome. For endometrial hyperplasia, use of pravastatin (adjusted HR, 2.50; 95% CI, 1.03–6.10) was significantly associated with increased risk of endometrial hyperplasia, whereas use of other types of statins was not significantly associated with risk of endometrial hyperplasia. For other outcomes, use of simvastatin, atorvastatin, rosuvastatin, or pravastatin was all not significantly associated with risk of ovarian cancer, endometrial cancer, ovarian cyst, endometriosis, endometrial polyp, or cervical polyp.

**Fig. 3:**
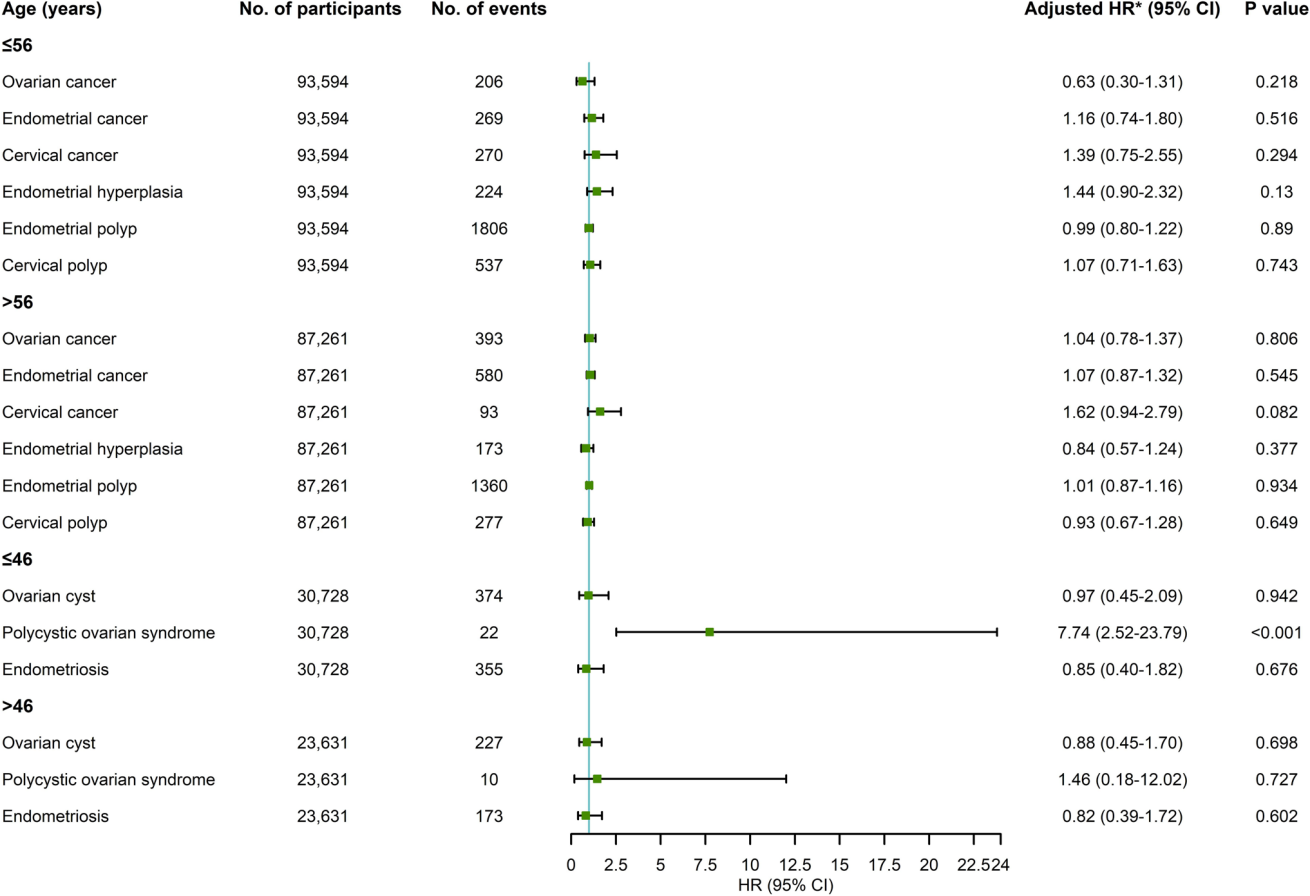
The associations between use of statins and risks of ovarian cancer, endometrial cancer, cervical cancer, ovarian cyst, polycystic ovarian syndrome, endometriosis, endometrial hyperplasia, endometrial polyp, and cervical polyp stratified by the median age. HR hazard ratio, CI confidence interval. *Adjusted for race, Townsend deprivation index, smoking status, alcohol use, vigorous physical activity, number of childbirth, number of abortion, any comorbidity at baseline (hyperlipidemia, ischemic heart disease, ischemic cerebrovascular disease, hypertension, diabetes, obesity, or pelvic inflammatory disease), oral contraceptive, and extra outcome-specific covariates. Moreover, the analyses of ovarian cyst, polycystic ovarian syndrome and endometriosis were restricted to the premenopausal female cohort

Figure 3 shows stratified analyses by the median age. There remained no significant association between use of statins and risk of ovarian cancer, endometrial cancer, ovarian cyst, endometriosis, endometrial hyperplasia, endometrial polyp, or cervical polyp in all age groups. A tendency toward increased risk of cervical cancer was observed in statin users aged > 56 years (adjusted HR, 1.62; 95% CI, 0.94–2.79), but this tendency was not observed in users aged ≤ 56 years (adjusted HR, 1.39; 95% CI, 0.75–2.55). Moreover, increased risk for polycystic ovarian syndrome from use of statins was seen in premenopausal females aged ≤ 46 years (adjusted HR, 7.74; 95% CI, 2.52–23.79), whereas no significant association was seen in premenopausal females aged > 46 years (adjusted HR, 1.46; 95% CI, 0.18–12.02).

**Fig. 4:**
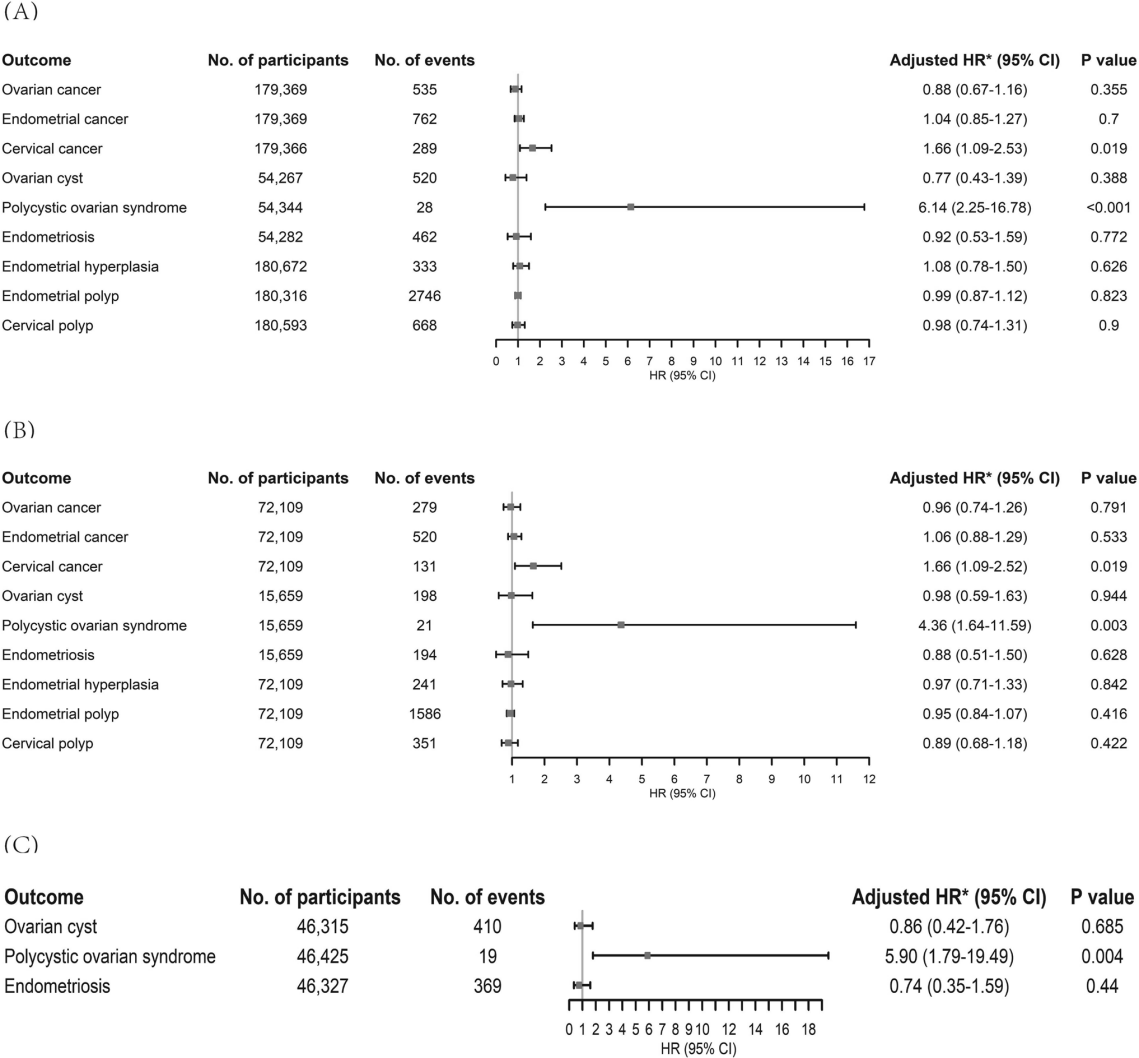
Sensitivity analyses for the associations between use of statins and risks of ovarian cancer, endometrial cancer, cervical cancer, ovarian cyst, polycystic ovarian syndrome, endometriosis, endometrial hyperplasia, endometrial polyp, and cervical polyp by excluding the first year of follow-up (**A**), restricting the study population to females with hyperlipidemia, ischemic heart disease, ischemic cerebrovascular disease, hypertension, diabetes, or obesity (**B**), and censoring the follow-up of premenopausal females at age 51 (**C**). HR hazard ratio, CI confidence interval. *Adjusted for age, race, Townsend deprivation index, smoking status, alcohol use, vigorous physical activity, number of childbirth, number of abortion, any comorbidity at baseline (hyperlipidemia, ischemic heart disease, ischemic cerebrovascular disease, hypertension, diabetes, obesity, or pelvic inflammatory disease), oral contraceptive, and extra outcome-specific covariates. Moreover, the analyses of ovarian cyst, polycystic ovarian syndrome, and endometriosis were restricted to the premenopausal female cohort

In our sensitivity analyses, the associations between use of statins and risks of all outcomes remained: (1) when we excluded the first year of follow-up (for all individuals) (Fig. 4A); (2) when we restricted the study population to females with hyperlipidemia, ischemic heart disease, ischemic cerebrovascular disease, hypertension, diabetes, or obesity (Fig. 4B); and (3) when we censored the follow-up of premenopausal females at age 51 (Fig. 4C).

## Discussion

### Principal findings

In this large-scale cohort study, we found that use of statins was significantly associated with increased risks of cervical cancer and polycystic ovarian syndrome, but was not significantly associated with risk of ovarian cancer, endometrial cancer, ovarian cyst, endometriosis, endometrial hyperplasia, endometrial polyp, or cervical polyp. We also noticed the potential modifying effects of statin type and age on the aforementioned associations. For instance, use of simvastatin was significantly associated with increased risk of polycystic ovarian syndrome and was borderline associated with increased risk of cervical cancer; use of atorvastatin was significantly associated with increased risk of polycystic ovarian syndrome; use of pravastatin was significantly associated with increased risks of cervical cancer and endometrial hyperplasia. Moreover, when we stratified by the median age, increased risk for polycystic ovarian syndrome from use of statins was only seen in premenopausal females aged ≤ 46 years.

### Compared with previous studies

The relationship between use of statins and risks of ovarian cancer and endometrial cancer is an intensely disputed topic. Some case–control studies found that use of statins was associated with reduced risks of ovarian cancer and endometrial cancer, which suggests that statins might have preventive effects on ovarian cancer and endometrial cancer [20, 21]. However, in recent years, more and more cohort and case–control studies showed that use of statins was not associated with risk of ovarian cancer or endometrial cancer [22–24]. Our study also found no association between use of statins and risk of ovarian cancer or endometrial cancer and does not support that use of statins may prevent ovarian cancer or endometrial cancer.

Our study indicated that use of statins was associated with increased risk of cervical cancer, which is inconsistent with a prior cohort study conducted by Kim et al. Kim et al.’s study is the only clinical study to date exploring the association between use of statins and risk of cervical cancer. That study used health insurance claims data and found that use of statins was associated with reduced risk of cervical cancer [22]. We cannot completely explain the discrepancies between Kim et al.’s study and our study, but it should be noted that some differences in study design exist. Due to the limited information contained in health insurance claims data, Kim et al.’s study only analyzed the potential confounding effects of age, comorbidities, and co-medication and was unable to analyze the potential confounding effects of other sociodemographic, lifestyle, and clinical factors. In our study, UK Biobank contains extensive sociodemographic, lifestyle, and clinical information. Thus, compared with Kim et al.’s study, we further analyzed the potential confounding effects of Townsend deprivation index, smoking status, alcohol use, vigorous physical activity, number of childbirth, number of abortion, lifetime number of sexual partners, age first had sexual intercourse, and oral contraceptive. All these factors have been reported to be correlated with the occurrence of cervical cancer. For example, socioeconomic deprivation, smoking, alcohol use, multiple sexual partners, early age at first intercourse, and use of oral contraceptives are important risk factors for cervical cancer [25–27], while multiple childbirth is a protective factor for cervical cancer [28]. By adjusting for these potential confounding factors, our study might provide more reliable results than Kim et al.’s study. Several possible mechanisms might explain the increased risk of cervical cancer associated with use of statins. First, inhibition of serum cholesterol levels by statins may be associated with increased risk of cancer [29]. Second, statins could enhance mitotic abnormalities, which may interfere with centromere development and function, leading to increased risk of mutations and cancer [30]. Third, statins could increase regulatory T cell numbers, which may impair the antitumor immune response of the host [31].

Our study also found that use of statins was associated with increased risk of polycystic ovarian syndrome and was not associated with risk of endometriosis. These findings are inconsistent with previous experimental studies of human cell lines and animal models. For example, previous experimental studies suggest that statins might prevent polycystic ovarian syndrome by reducing steroid hormone synthesis and inhibiting the growth of theca-interstitial cells in ovaries [4, 5]. In addition, previous experimental studies also suggest that statins might prevent endometriosis due to their antiproliferative and pro-apoptotic effects on endometrial and endometriotic cells, their ability to reduce cell viability and migration, the inhibition of angiogenesis, and anti-inflammatory activities [5, 6]. As it is possible that the effects of statins in patients may be different from those observed in cell culture or animal models, our cohort study provides more credible results than previous experimental studies.

### Explain unexpected findings

For cervical cancer, when we performed subgroup analysis stratified by statin type or the median age, the association was attenuated in most subgroups, which may be due to the decreased sample size. However, the association with cervical cancer risk was enhanced in the pravastatin subgroup. Similarly, some previous clinical studies also found that pravastatin was more likely to increase cancer risk than other types of statins. For example, a cohort study by Desai et al. indicated that use of pravastatin was associated with increased risk of ovarian cancer, whereas use of other types of statins was not [32]. In addition, a record-linkage study by Haukka et al. showed that use of pravastatin was associated with increased risk of non-melanoma skin cancer, whereas use of other types of statins was not [33]. The mechanism why pravastatin is more likely to increase cancer risk than other types of statins is unclear, but may be related to the highly hydrophilic property of pravastatin. Based on their solubility, statins can be chemically classified as lipophilic statins and hydrophilic statins. Lipophilic statins enter cells through passive diffusion, whereas hydrophilic statins enter cells through active transport. It is postulated that the cellular uptake pattern of statins might be related to their effect on tumor growth [32].

For polycystic ovarian syndrome, we found that increased risk for polycystic ovarian syndrome from use of statins was seen in premenopausal females aged ≤ 46 years, whereas no significant association was seen in premenopausal females aged > 46 years. Polycystic ovarian syndrome mainly occurs in reproductive aged females (12–45 years) and is less likely to occur in females aged > 46 years [34]. Similarly, in our study, only 10 polycystic ovarian syndrome cases occurred in premenopausal females aged > 46 years, and the result for this subgroup was imprecise (with wide confidence interval) and may be a false negative. Thus, further studies are needed to verify this finding and explore the underlying mechanism.

For endometrial hyperplasia, we found that use of pravastatin was significantly associated with increased risk of endometrial hyperplasia, whereas use of other types of statins was not. Currently, there is no clear explanation for this finding. Besides, as the sample size of pravastatin users (664) was relatively small, we could not rule out the possibility that the increased risk for endometrial hyperplasia from use of pravastatin was due to chance. Thus, further studies are also needed to confirm this finding and explore the possible mechanism.

### Strengths and limitations

Our study has several strengths. First, the UK Biobank contains extensive sociodemographic, lifestyle, and clinical information, which enabled us to adjust for a wide range of confounders and conduct multiple subgroup analyses. Second, most subgroup and sensitivity analyses showed consistent results with the main analysis, which further confirmed the robustness of our results. Third, the prospective design limited recall bias on the assessment of statins.

Our study also has some limitations. First, use of statins and some covariates were assessed by self-report, which might induce misclassification. Such misclassification is likely non-differential between individuals with and without outcome events, which would attenuate the association toward null. However, this cannot flip a protective effect (HR < 1) to a harmful one (HR > 1). Second, we did not have information on duration or dosage of statins, and it may take time for statins to have effects on outcome events. Further studies are needed to evaluate the impacts of these factors on results. Third, the diagnosis information of ovarian cyst, polycystic ovarian syndrome, endometriosis, endometrial hyperplasia, endometrial polyp, and cervical polyp was obtained by linking to hospital inpatient data. That said, these diseases diagnosed at the outpatient clinic and asymptomatic/undiagnosed ones were not captured in our data. This misclassification might be differential between statin users and non-users because users have more frequent healthcare visits and are subject to surveillance bias. Fourth, potential reverse causality may exist in our study as it takes years for outcome events to develop. However, the results remained unchanged when we excluded the first year of follow-up. Fifth, although we adjusted for all main indications for statins in the statistical model and further performed sensitivity analysis, indication bias could not be completely avoided.

## Conclusions and clinical and research implications

In conclusion, in this cohort study of UK Biobank female participants, use of statins was associated with increased risks of cervical cancer and polycystic ovarian syndrome, but was not associated with increased or decreased risk of ovarian cancer, endometrial cancer, ovarian cyst, endometriosis, endometrial polyp, or cervical polyp. Unlike some previous studies, our findings do not support that use of statins may prevent ovarian cancer, endometrial cancer, cervical cancer, polycystic ovarian syndrome, or endometriosis. Moreover, according to our findings, the potential risks of cervical cancer and polycystic ovarian syndrome associated with use of statins are of great importance and should be closely monitored in future clinical practice. However, our findings should be interpreted with cautions due to indication and surveillance biases.

### Supplementary Information

Below is the link to the electronic supplementary material.

Supplementary file1 (DOCX 53 KB)

## Data Availability

Data from the UK Biobank (http://www.ukbiobank.ac.uk/) are available to all researchers upon making an application. Part of this research was conducted using the UK Biobank Resource under Application 54803.

## Declarations

### Disclaimer

The funders had no role in the development of this article (i.e., in the study design; collection, analysis, and interpretation of data; report writing; or decision to submit the paper for publication).

## Abbreviations

HMG-CoA: 3-Hydroxy-3-methyl-glutaryl coenzyme A
FAERS: FDA Adverse Event Reporting System
HES: Hospital Episode Statistics for England
SMR: Scottish Morbidity Record
PEDW: Patient Episode Database for Wales
NHS: National Health Service
SD: Standard deviation
IQR: Interquartile range
HRs: Hazard ratios
95% CI: 95% Confidence intervals

## References

[1] Wong ND, Young D, Zhao Y, Nguyen H, Caballes J, Khan I (2016). Prevalence of the American College of Cardiology/American Heart Association statin eligibility groups, statin use, and low-density lipoprotein cholesterol control in US adults using the National Health and Nutrition Examination Survey 2011–2012. J Clin Lipidol.

[2] Vancheri F, Backlund L, Strender LE, Godman B, Wettermark B (2016). Time trends in statin utilisation and coronary mortality in Western European countries. BMJ Open.

[3] Adedinsewo D, Taka N, Agasthi P, Sachdeva R, Rust G, Onwuanyi A (2016). Prevalence and factors associated with statin use among a nationally representative sample of US adults: National Health and Nutrition Examination Survey, 2011–2012. Clin Cardiol.

[4] De La Cruz JA, Mihos CG, Horvath SA, Santana O (2019). The pleiotropic effects of statins in endocrine disorders. Endocr Metab Immune Disord Drug Targets.

[5] Zeybek B, Costantine M, Kilic GS, Borahay MA (2018). Therapeutic roles of statins in gynecology and obstetrics: the current evidence. Reprod Sci.

[6] Vitagliano A, Noventa M, Quaranta M, Gizzo S (2016). Statins as targeted “magical pills” for the conservative treatment of endometriosis: may potential adverse effects on female fertility represent the “dark side of the same coin”? A systematic review of literature. Reprod Sci.

[7] William GP, Suhail M, Jaferi F, Nasim M (2014). Effects of simvastatin 20mg on the histology of albino rat ovary. Pakistan Journal of Medical and Health Sciences.

[8] Jiao XF, Li HL, Jiao XY, Guo YC, Zhang C, Yang CS (2020). Ovary and uterus related adverse events associated with statin use: an analysis of the FDA Adverse Event Reporting System. Sci Rep.

[9] Duggirala HJ, Tonning JM, Smith E, Bright RA, Baker JD, Ball R (2016). Use of data mining at the Food and Drug Administration. J Am Med Inform Assoc.

[10] Sudlow C, Gallacher J, Allen N, Beral V, Burton P, Danesh J (2015). UK biobank: an open access resource for identifying the causes of a wide range of complex diseases of middle and old age. PLoS Med.

[11] Jiao X, Li H, Zeng L, Han L, Yang H, Hu Y (2023). Use of statins and risk of uterine leiomyoma: a cohort study in the UK Biobank. J Evid Based Med.

[12] UK Biobank (2021) Touch Screen Questionnaire Version 1.0. https://biobank.ndph.ox.ac.uk/showcase/refer.cgi?id=100247. Accessed 18 Sep 2022

[13] UK Biobank (2020) Data providers and dates of data availability. https://biobank.ndph.ox.ac.uk/showcase/exinfo.cgi?src=Data_providers_and_dates. Accessed 18 Sep 2022

[14] Liu Z, Luo Y, Ren J, Yang L, Li J, Wei Z (2022). Association between fish oil supplementation and cancer risk according to fatty fish consumption: a large prospective population-based cohort study using UK Biobank. Int J Cancer.

[15] UK Biobank. The verbal interview within ACE centres. 2012. https://biobank.ndph.ox.ac.uk/showcase/ukb/docs/Interview.pdf. Accessed 18 Sep 2022).

[16] Knuppel A, Papier K, Fensom GK, Appleby PN, Schmidt JA, Tong TYN (2020). Meat intake and cancer risk: prospective analyses in UK Biobank. Int J Epidemiol.

[17] Blane D (1989). Health and deprivation-inequality and the North-Townsend, P, Phillimore, P, Beattie. A Brit J Sociol.

[18] Sarri G, Davies M, Lumsden MA, Guideline DG (2015). Diagnosis and management of menopause: summary of NICE guidance. BMJ.

[19] Yaddanapudi LN (2016). The American Statistical Association statement on P-values explained. J Anaesthesiol Clin Pharmacol.

[20] Akinwunmi B, Vitonis AF, Titus L, Terry KL, Cramer DW (2019). Statin therapy and association with ovarian cancer risk in the New England Case Control (NEC) study. Int J Cancer.

[21] Lavie O, Pinchev M, Rennert HS, Segev Y, Rennert G (2013). The effect of statins on risk and survival of gynecological malignancies. Gynecol Oncol.

[22] Kim DS, Ahn HS, Kim HJ (2022). Statin use and incidence and mortality of breast and gynecology cancer: a cohort study using the National Health Insurance claims database. Int J Cancer.

[23] Sperling CD, Verdoodt F, Friis S, Dehlendorff C, Kjaer SK (2017). Statin use and risk of endometrial cancer: a nationwide registry-based case-control study. Acta Obstet Gynecol Scand.

[24] Baandrup L, Dehlendorff C, Friis S, Olsen JH, Kjaer SK (2015). Statin use and risk for ovarian cancer: a Danish nationwide case-control study. Br J Cancer.

[25] Huang J, Deng Y, Boakye D, Tin MS, Lok V, Zhang L (2022). Global distribution, risk factors, and recent trends for cervical cancer: a worldwide country-level analysis. Gynecol Oncol.

[26] Zhang S, Xu H, Zhang L, Qiao Y (2020). Cervical cancer: epidemiology, risk factors and screening. Chin J Cancer Res.

[27] Srivastava S, Shahi UP, Dibya A, Gupta S, Roy JK (2014). Distribution of HPV genotypes and involvement of risk factors in cervical lesions and invasive cervical cancer: a study in an Indian population. Int J Mol Cell Med.

[28] Makuza JD, Nsanzimana S, Muhimpundu MA, Pace LE, Ntaganira J, Riedel DJ (2015). Prevalence and risk factors for cervical cancer and pre-cancerous lesions in Rwanda. Pan Afr Med J.

[29] Gonyeau MJ, Yuen DW (2010). A clinical review of statins and cancer: helpful or harmful?. Pharmacotherapy.

[30] Lamprecht J, Wójcik C, Jakóbisiak M, Stoehr M, Schrorter D, Paweletz N (1999). Lovastatin induces mitotic abnormalities in various cell lines. Cell Biol Int.

[31] Fujimoto M, Higuchi T, Hosomi K, Takada M (2015). Association between statin use and cancer: data mining of a spontaneous reporting database and a claims database. Int J Med Sci.

[32] Desai P, Wallace R, Anderson ML, Howard BV, Ray RM, Wu C (2018). An analysis of the association between statin use and risk of endometrial and ovarian cancers in the Women’s Health Initiative. Gynecol Oncol.

[33] Haukka J, Sankila R, Klaukka T, Lonnqvist J, Niskanen L, Tanskanen A (2010). Incidence of cancer and statin usage–record linkage study. Int J Cancer.

[34] Lamba P, Sharma D, Sinnarkar VV (2022). Polycystic ovarian syndrome treated with individualized homeopathy: a case report. Altern Ther Health Med.

