# Supplementary material for: Use of statins and risks of ovarian, uterine, and cervical diseases: a cohort study in the UK Biobank
Source: Eur J Clin Pharmacol. 2024 Feb 28;80(6):855–67. doi: 10.1007/s00228-024-03656-y (PMC11098902; doi:10.1007/s00228-024-03656-y)
Supplement: Supplementary file 1 — Supplementary file1 (DOCX 53 KB) [file 228_2024_3656_MOESM1_ESM.docx]

**List of supplementary Tables**

**Supplemental Table 1.** The variable names, data-fields and data-coding of inclusion and exclusion criteria.

**Supplemental Table 2.** The data-fields and data-coding of statins.

**Supplemental Table 3.** The variable names, data-fields and data-coding of the outcome.

**Supplemental Table 4.** Extra related covariates for each outcome.

**Supplemental Table 5.** The variable names, data-fields and data-coding of covariates.

**Supplemental Table 6.** Baseline characteristics of premenopausal females by use of statins.

**SUPPLEMENTAL TABLE 1**

**The variable names, data-fields and data-coding of inclusion and exclusion criteria**

| **Inclusion and exclusion criteria** | **Variable name** | **Data-field** | **Data-coding** |
| --- | --- | --- | --- |
| Female | Female | Baseline characteristics - Population characteristics:  Sex (31) | 0 |
| Cancer | Malignant neoplasm;  In situ neoplasms | Summary Diagnoses - Hospital inpatient - Health-related outcomes:  Diagnoses - ICD10 (41270),  Date of first in-patient diagnosis - ICD10 (41280),  Diagnoses - ICD9 (41271),  Date of first in-patient diagnosis - ICD9 (41281) | ICD10: C%, D0%;  ICD9: 14%-20%, 230-234, 230%-234% |
|  | Carcinoma in situ;  Malignant;  Malignant, primary site;  Malignant, metastatic site;  Malignant, uncertain whether primary or metastatic site;  Malignant, microinvasive | Cancer register - Health-related outcomes:  Date of cancer diagnosis (40005),  Behaviour of cancer tumour (40012) | 2, -1, 3, 6, 9, 5 |
|  |  | Medical conditions - Verbal interview - UK Biobank Assessment Centre:  Cancer code, self-reported (20001) | Non-null value |
| Nonmelanoma skin cancer | Nonmelanoma skin cancer | Summary Diagnoses - Hospital inpatient - Health-related outcomes:  Diagnoses - ICD10 (41270),  Date of first in-patient diagnosis - ICD10 (41280),  Diagnoses - ICD9 (41271),  Date of first in-patient diagnosis - ICD9 (41281) | ICD10: C44, C44%；  ICD9: 173, 173% |
|  | Nonmelanoma skin cancer | Cancer register - Health-related outcomes:  Type of cancer: ICD10 (40006),  Type of cancer: ICD9 (40013),  Date of cancer diagnosis (40005) | ICD10: C44, C44%；  ICD9: 173, 173% |
|  | Non-melanoma skin cancer;  Basal cell carcinoma;  Squamous cell carcinoma;  Rodent ulcer | Medical conditions - Verbal interview - UK Biobank Assessment Centre：  Cancer code, self-reported (20001) | 1060, 1061, 1062, 1073 |
| Ovarian cyst | Follicular cyst of ovary;  Corpus luteum cyst;  Other and unspecified ovarian cysts;  Developmental ovarian cyst | Summary Diagnoses - Hospital inpatient - Health-related outcomes:  Diagnoses - ICD10 (41270),  Date of first in-patient diagnosis - ICD10 (41280),  Diagnoses - ICD9 (41271),  Date of first in-patient diagnosis - ICD9 (41281) | ICD10: N830-N832, Q501;  ICD9: 6200-6202 |
|  | Ovarian cyst or cysts | Medical conditions - Verbal interview - UK Biobank Assessment Centre：  Non-cancer illness code, self-reported (20002) | 1349 |
| Polycystic ovarian syndrome | Polycystic ovarian syndrome;  Polycystic ovaries | Summary Diagnoses - Hospital inpatient - Health-related outcomes:  Diagnoses - ICD10 (41270),  Date of first in-patient diagnosis - ICD10 (41280),  Diagnoses - ICD9 (41271),  Date of first in-patient diagnosis - ICD9 (41281) | ICD10: E282;  ICD9: 2564 |
|  | Polycystic ovaries / polycystic ovarian syndrome | Medical conditions - Verbal interview - UK Biobank Assessment Centre：  Non-cancer illness code, self-reported (20002) | 1350 |
| Endometriosis | Endometriosis | Summary Diagnoses - Hospital inpatient - Health-related outcomes:  Diagnoses - ICD10 (41270),  Date of first in-patient diagnosis - ICD10 (41280),  Diagnoses - ICD9 (41271),  Date of first in-patient diagnosis - ICD9 (41281) | ICD10: N80, N80%;  ICD9: 617, 617% |
|  | Endometriosis | Medical conditions - Verbal interview - UK Biobank Assessment Centre：  Non-cancer illness code, self-reported (20002) | 1402 |
| Endometrial hyperplasia | Endometrial hyperplasia;  Endometrial glandular hyperplasia;  Endometrial adenomatous hyperplasia;  Endometrial cystic hyperplasia | Summary Diagnoses - Hospital inpatient - Health-related outcomes:  Diagnoses - ICD10 (41270),  Date of first in-patient diagnosis - ICD10 (41280),  Diagnoses - ICD9 (41271),  Date of first in-patient diagnosis - ICD9 (41281) | ICD10: N850, N851;  ICD9: 6213 |
| Endometrial polyp | Polyp of corpus uteri | Summary Diagnoses - Hospital inpatient - Health-related outcomes:  Diagnoses - ICD10 (41270),  Date of first in-patient diagnosis - ICD10 (41280),  Diagnoses - ICD9 (41271),  Date of first in-patient diagnosis - ICD9 (41281) | ICD10: N840;  ICD9: 6210 |
|  | Uterine polyps | Medical conditions - Verbal interview - UK Biobank Assessment Centre：  Non-cancer illness code, self-reported (20002) | 1352 |
| Cervical polyp | Polyp of cervix uteri;  Mucous polyp of cervix | Summary Diagnoses - Hospital inpatient - Health-related outcomes:  Diagnoses - ICD10 (41270),  Date of first in-patient diagnosis - ICD10 (41280),  Diagnoses - ICD9 (41271),  Date of first in-patient diagnosis - ICD9 (41281) | ICD10: N841;  ICD9: 6227 |
|  | Cervical polyps | Medical conditions - Verbal interview - UK Biobank Assessment Centre：  Non-cancer illness code, self-reported (20002) | 1555 |
| Ovariectomy | Partial excision of ovary;  Bilateral oophorectomy NEC;  Unilateral oophorectomy NEC;  Oophorectomy of remaining solitary ovary NEC;  Endoscopic extirpation of lesion of ovary NEC;  Partial oophorectomy;  Partial oophorectomy: wedge resection;  Partial oophorectomy: excision of lesion;  Extirpation of lesion of ovarian region, not elsewhere classified;  Salpingo-oophorectomy: unilateral;  Salpingo-oophorectomy: bilateral | Summary Operations - Hospital inpatient - Health-related outcomes:  Operative procedures - OPCS4 (41272),  Date of first operative procedure - OPCS4 (41282),  Operative procedures - OPCS3 (41273),  Date of first operative procedure - OPCS3 (41283) | OPCS4: Q43, Q43%, Q223, Q235, Q236, Q491;  OPCS3: 672, 672%, 673, 681, 6811, 6812 |
|  | Bilateral oophorectomy;  Unilateral oophorectomy;  Ovarian cyst removal/surgery | Operations - Verbal interview - UK Biobank Assessment Centre:  Operation code (20004) | 1355, 1356, 1506 |
|  | Bilateral oophorectomy (both ovaries removed) | Female-specific factors - Sex-specific factors - Touchscreen - UK Biobank Assessment Centre：  Bilateral oophorectomy (both ovaries removed) (2834) | 1 |
| Hysterectomy | Abdominal excision of uterus;  Vaginal excision of uterus;  Open myomectomy;  Open excision of lesion of uterus NEC;  Vaginal excision of lesion of uterus;  Endoscopic resection of lesion of uterus;  Extended hysterectomy;  Radical hysterectomy;  Radical hysterectomy: with pelvic lymphadenectomy;  Vaginal hysterectomy: total;  Vaginal hysterectomy: with repair of prolapse;  Sub-total hysterectomy;  Hysterectomy, not elsewhere classified;  Hysterectomy, not elsewhere classified: total hysterectomy;  Excision of lesion of uterus: myomectomy;  Excision of lesion of uterus: polypectomy | Summary Operations - Hospital inpatient - Health-related outcomes:  Operative procedures - OPCS4 (41272),  Date of first operative procedure - OPCS4 (41282),  Operative procedures - OPCS3 (41273),  Date of first operative procedure - OPCS3 (41283) | OPCS4: Q07, Q07%, Q08, Q08%, Q092, Q093, Q161, Q171;  OPCS3: 691, 692, 6921, 693, 693_, 694, 696, 6961, 700, 7001, 7002 |
|  | Hysterectomy;  Hysterectomy with oophorectomy;  Hysterectomy with cervical sparing;  Myomectomy/fibroids removed;  Uterine polypectomy/uterine polyps removed | Operations - Verbal interview - UK Biobank Assessment Centre:  Operation code (20004) | 1357, 1358, 1359, 1509, 1539 |
|  | Ever had hysterectomy (womb removed) | Female-specific factors - Sex-specific factors - Touchscreen - UK Biobank Assessment Centre：  Ever had hysterectomy (womb removed) (3591) | 1 |
| Cervicectomy | Excision of cervix uteri;  Excision of lesion of cervix;  Other operations on uterus: amputation of cervix | Summary Operations - Hospital inpatient - Health-related outcomes:  Operative procedures - OPCS4 (41272),  Date of first operative procedure - OPCS4 (41282),  Operative procedures - OPCS3 (41273),  Date of first operative procedure - OPCS3 (41283) | OPCS4: Q01, Q01%;  OPCS3: 705, 705%, 7094 |
|  | Cervical polyps removed | Operations - Verbal interview - UK Biobank Assessment Centre:  Operation code (20004) | 1541 |
| Menopause | Menopause | Female-specific factors - Sex-specific factors - Touchscreen - UK Biobank Assessment Centre:  Had menopause (2724) | Yes: 1;  No: 0;  Missing: 2, 3, -3, NA |

ICD10: International Classification of Diseases, 10th Revision; ICD9: International Classification of Diseases, 9th revision; OPCS4: Office of Population Censuses and Surveys Classification of Interventions and Procedures, 4th Revision; OPCS3: Office of Population Censuses and Surveys Classification of Interventions and Procedures, 3th revision; %: represent any number of characters; _: represent a single character.

**SUPPLEMENTAL TABLE 2**

**The data-fields and data-coding of statins**

| **Statin** | **Data-field** | **Data-coding** |
| --- | --- | --- |
| Simvastatin | Medications - Verbal interview - UK Biobank Assessment Centre：  Treatment/medication code (20003) | 1140861958 |
| Atorvastatin | Medications - Verbal interview - UK Biobank Assessment Centre：  Treatment/medication code (20003) | 1141146234 |
| Rosuvastatin | Medications - Verbal interview - UK Biobank Assessment Centre：  Treatment/medication code (20003) | 1141192410 |
| Pravastatin | Medications - Verbal interview - UK Biobank Assessment Centre：  Treatment/medication code (20003) | 1140888648 |

**SUPPLEMENTAL TABLE 3**

**The variable names, data-fields and data-coding of the outcome**

| **Outcome** | **Variable name** | **Data-field** | **Data-coding** |
| --- | --- | --- | --- |
| Ovarian cancer | Malignant neoplasm of ovary | Cancer register - Health-related outcomes:  Type of cancer: ICD10 (40006),  Type of cancer: ICD9 (40013),  Date of cancer diagnosis (40005) | ICD10: C56;  ICD9: 1830 |
|  | Malignant neoplasm of ovary | Summary Diagnoses - Hospital inpatient - Health-related outcomes:  Diagnoses - ICD10 (41270),  Date of first in-patient diagnosis - ICD10 (41280),  Diagnoses - ICD9 (41271),  Date of first in-patient diagnosis - ICD9 (41281) | ICD10: C56;  ICD9: 1830 |
| Endometrial cancer | Malignant neoplasm of corpus uteri, endometrium;  Carcinoma in situ of other and unspecified genital organs, endometrium | Cancer register - Health-related outcomes:  Type of cancer: ICD10 (40006),  Type of cancer: ICD9 (40013),  Date of cancer diagnosis (40005) | ICD10: C541, D070 |
|  | Malignant neoplasm of corpus uteri, endometrium;  Carcinoma in situ of other and unspecified genital organs, endometrium | Summary Diagnoses - Hospital inpatient - Health-related outcomes:  Diagnoses - ICD10 (41270),  Date of first in-patient diagnosis - ICD10 (41280),  Diagnoses - ICD9 (41271),  Date of first in-patient diagnosis - ICD9 (41281) | ICD10: C541, D070 |
| Cervical cancer | Malignant neoplasm of cervix uteri;  Carcinoma in situ of cervix uteri | Cancer register - Health-related outcomes:  Type of cancer: ICD10 (40006),  Type of cancer: ICD9 (40013),  Date of cancer diagnosis (40005) | ICD10: C53, C53%, D06, D06%;  ICD9: 180, 180%, 2331 |
|  | Malignant neoplasm of cervix uteri;  Carcinoma in situ of cervix uteri | Summary Diagnoses - Hospital inpatient - Health-related outcomes:  Diagnoses - ICD10 (41270),  Date of first in-patient diagnosis - ICD10 (41280),  Diagnoses - ICD9 (41271),  Date of first in-patient diagnosis - ICD9 (41281) | ICD10: C53, C53%, D06, D06%;  ICD9: 180, 180%, 2331 |
| Ovarian cyst | Follicular cyst of ovary;  Corpus luteum cyst;  Other and unspecified ovarian cysts;  Developmental ovarian cyst | Summary Diagnoses - Hospital inpatient - Health-related outcomes:  Diagnoses - ICD10 (41270),  Date of first in-patient diagnosis - ICD10 (41280),  Diagnoses - ICD9 (41271),  Date of first in-patient diagnosis - ICD9 (41281) | ICD10: N830-N832, Q501;  ICD9: 6200-6202 |
| Polycystic ovarian syndrome | Polycystic ovarian syndrome;  Polycystic ovaries | Summary Diagnoses - Hospital inpatient - Health-related outcomes:  Diagnoses - ICD10 (41270),  Date of first in-patient diagnosis - ICD10 (41280),  Diagnoses - ICD9 (41271),  Date of first in-patient diagnosis - ICD9 (41281) | ICD10: E282;  ICD9: 2564 |
| Endometriosis | Endometriosis | Summary Diagnoses - Hospital inpatient - Health-related outcomes:  Diagnoses - ICD10 (41270),  Date of first in-patient diagnosis - ICD10 (41280),  Diagnoses - ICD9 (41271),  Date of first in-patient diagnosis - ICD9 (41281) | ICD10: N80, N80%;  ICD9: 617, 617% |
| Endometrial hyperplasia | Endometrial hyperplasia;  Endometrial glandular hyperplasia;  Endometrial adenomatous hyperplasia;  Endometrial cystic hyperplasia | Summary Diagnoses - Hospital inpatient - Health-related outcomes:  Diagnoses - ICD10 (41270),  Date of first in-patient diagnosis - ICD10 (41280),  Diagnoses - ICD9 (41271),  Date of first in-patient diagnosis - ICD9 (41281) | ICD10: N850, N851;  ICD9: 6213 |
| Endometrial polyp | Polyp of corpus uteri | Summary Diagnoses - Hospital inpatient - Health-related outcomes:  Diagnoses - ICD10 (41270),  Date of first in-patient diagnosis - ICD10 (41280),  Diagnoses - ICD9 (41271),  Date of first in-patient diagnosis - ICD9 (41281) | ICD10: N840;  ICD9: 6210 |
| Cervical polyp | Polyp of cervix uteri;  Mucous polyp of cervix | Summary Diagnoses - Hospital inpatient - Health-related outcomes:  Diagnoses - ICD10 (41270),  Date of first in-patient diagnosis - ICD10 (41280),  Diagnoses - ICD9 (41271),  Date of first in-patient diagnosis - ICD9 (41281) | ICD10: N841;  ICD9: 6227 |

ICD10: International Classification of Diseases, 10th Revision; ICD9: International Classification of Diseases, 9th revision; %: represent any number of characters.

**SUPPLEMENTAL TABLE 4**

**Extra related covariates for each outcome**

| **Outcome** | **Additionally adjusted covariates** |
| --- | --- |
| **Ovarian cancer** | Age at menarche, age at first birth, HRT |
| **Endometrial cancer** | Menopause, HRT, tamoxifen |
| **Cervical cancer** | Lifetime number of sexual partners, age first had sexual intercourse (<16 or ≥16 years), any comorbidity at baseline (HPV infection or HIV disease) |
| **Ovarian cyst** | — |
| **Polycystic ovarian syndrome** | Age at menarche |
| **Endometriosis** | Age at menarche, age at first birth |
| **Endometrial hyperplasia** | Tamoxifen |
| **Endometrial polyp** | Menopause, HRT, tamoxifen |
| **Cervical polyp** | Menopause, HRT, comorbidity at baseline (HPV infection) |

HRT, hormone-replacement therapy; HPV, human papillomavirus; HIV, Human immunodeficiency virus; —, none.

**SUPPLEMENTAL TABLE 5**

**The variable names, data-fields and data-coding of covariates**

| **Covariates** | **Variable name** | **Data-field** | **Data-coding** |
| --- | --- | --- | --- |
| Age | Age | Baseline characteristics - Population characteristics:  Age at recruitment (21022) | / |
| Race | Ethnicity | Ethnicity - Sociodemographics - Touchscreen - UK Biobank Assessment Centre:  Ethnic background (21000) | White: 1, 100%;  Others: 2, 200%, 3, 300%, 4, 400%, 5, 6;  Missing: -1, -3, NA |
| Townsend deprivation index | Townsend deprivation index | Baseline characteristics - Population characteristics:  Townsend deprivation index at recruitment (189) | / |
| Smoking status | Smoking status | Smoking - Lifestyle and environment - Touchscreen - UK Biobank Assessment Centre:  Smoking status (20116) | Never: 0;  Past: 1;  Current: 2；  Missing: -3, NA |
| Alcohol use | Alcohol intake frequency | Alcohol - Lifestyle and environment - Touchscreen - UK Biobank Assessment Centre:  Alcohol intake frequency (1558) | Daily or almost daily: 1;  Three or four times a week: 2;  Once or twice a week: 3；  One to three times a month: 4;  Special occasions only: 5;  Never: 6;  Missing: -3, NA |
| Vigorous physical activity | Vigorous physical activity | MET Scores - Physical activity - Lifestyle and environment - Touchscreen - UK Biobank Assessment Centre：  IPAQ activity group (22032) | Low: 0;  Moderate: 1;  High: 2；  Missing: NA |
| Number of childbirth | Number of live births;  Number of stillbirths | Female-specific factors - Sex-specific factors - Touchscreen - UK Biobank Assessment Centre:  Number of live births (2734),  Number of stillbirths (3829) | / |
| Number of abortion | Number of spontaneous miscarriages;  Number of pregnancy terminations | Female-specific factors - Sex-specific factors - Touchscreen - UK Biobank Assessment Centre:  Number of spontaneous miscarriages (3839),  Number of pregnancy terminations (3849),  Ever had stillbirth, spontaneous miscarriage or termination (2774) | / |
| Age at menarche | Age when periods started (menarche) | Female-specific factors - Sex-specific factors - Touchscreen - UK Biobank Assessment Centre:  Age when periods started (menarche) (2714) | / |
| Age at first birth | Age at first live birth | Female-specific factors - Sex-specific factors - Touchscreen - UK Biobank Assessment Centre:  Age at first live birth (2754) | / |
|  | Age of primiparous women at birth of child | Female-specific factors - Sex-specific factors - Touchscreen - UK Biobank Assessment Centre:  Age of primiparous women at birth of child (3872) | / |
| Menopause | Menopause | Female-specific factors - Sex-specific factors - Touchscreen - UK Biobank Assessment Centre:  Had menopause (2724) | Yes: 1;  No: 0；  Missing: 2, 3, -3, NA |
| Lifetime number of sexual partners | Lifetime number of sexual partners | Sexual factors - Lifestyle and environment - Touchscreen - UK Biobank Assessment Centre:  Lifetime number of sexual partners (2149) | / |
| Age first had sexual intercourse | Age first had sexual intercourse | Sexual factors - Lifestyle and environment - Touchscreen - UK Biobank Assessment Centre：  Age first had sexual intercourse (2139) | / |
| Hyperlipidemia | Disorders of lipoprotein metabolism and other lipidaemias;  Pure hypercholesterolaemia;  Pure hyperglyceridaemia;  Mixed hyperlipidaemia;  Hyperchylomicronaemia;  Other hyperlipidaemia;  Hyperlipidaemia, unspecified;  Lipoprotein deficiency;  Other disorders of lipoprotein metabolism;  Disorder of lipoprotein metabolism, unspecified;  Disorders of lipoid metabolism;  Other and unspecified hyperlipidaemia;  Lipodystrophy | Summary Diagnoses - Hospital inpatient - Health-related outcomes:  Diagnoses - ICD10 (41270),  Date of first in-patient diagnosis - ICD10 (41280),  Diagnoses - ICD9 (41271),  Date of first in-patient diagnosis - ICD9 (41281) | ICD10: E78, E78%;  ICD9: 272, 272% |
|  | High cholesterol | Medical conditions - Verbal interview - UK Biobank Assessment Centre：  Non-cancer illness code, self-reported (20002) | 1473 |
| Ischaemic heart disease | Ischaemic heart diseases;  Angina pectoris;  Acute myocardial infarction;  Subsequent myocardial infarction;  Certain current complications following acute myocardial infarction;  Other acute ischaemic heart diseases;  Chronic ischaemic heart disease | Summary Diagnoses - Hospital inpatient - Health-related outcomes:  Diagnoses - ICD10 (41270),  Date of first in-patient diagnosis - ICD10 (41280),  Diagnoses - ICD9 (41271),  Date of first in-patient diagnosis - ICD9 (41281) | ICD10: I20-I25, I20%-I25%;  ICD9: 410-414, 4109, 4119, 4129, 4139, 414% |
|  | Heart attack;  Angina | Medical conditions - Health and medical history - Touchscreen - UK Biobank Assessment Centre：  Vascular/heart problems diagnosed by doctor (6150) | 1, 2 |
|  | Angina;  Heart attack/myocardial infarction | Medical conditions - Verbal interview - UK Biobank Assessment Centre：  Non-cancer illness code, self-reported (20002) | 1074, 1075 |
| Ischaemic cerebrovascular disease | Transient cerebral ischaemic attacks and related syndromes;  Cerebral infarction;  Stroke, not specified as haemorrhage or infarction;  Occlusion and stenosis of precerebral arteries, not resulting in cerebral infarction;  Occlusion and stenosis of cerebral arteries, not resulting in cerebral infarction;  Occlusion and stenosis of precerebral arteries;  Occlusion of cerebral arteries;  Transient cerebral ischaemia | Summary Diagnoses - Hospital inpatient - Health-related outcomes:  Diagnoses - ICD10 (41270),  Date of first in-patient diagnosis - ICD10 (41280),  Diagnoses - ICD9 (41271),  Date of first in-patient diagnosis - ICD9 (41281) | ICD10: G45, G45%, I63-I66, I63%-I66%;  ICD9: 433-435, 433%, 434%, 4359 |
|  | Stroke | Medical conditions - Health and medical history - Touchscreen - UK Biobank Assessment Centre：  Vascular/heart problems diagnosed by doctor (6150) | 3 |
|  | Ischaemic stroke;  Transient ischaemic attack (tia) | Medical conditions - Verbal interview - UK Biobank Assessment Centre：  Non-cancer illness code, self-reported (20002) | 1583, 1082 |
| Hypertension | Hypertensive diseases;  Essential (primary) hypertension;  Hypertensive heart disease;  Hypertensive renal disease;  Hypertensive heart and renal disease;  Secondary hypertension | Summary Diagnoses - Hospital inpatient - Health-related outcomes:  Diagnoses - ICD10 (41270),  Date of first in-patient diagnosis - ICD10 (41280),  Diagnoses - ICD9 (41271),  Date of first in-patient diagnosis - ICD9 (41281) | ICD10: I10, I11, I12, I13, I15, I10%, I11%, I12%, I13%, I15%  ICD9: 401-405, 401%-405% |
|  | High blood pressure | Medical conditions - Health and medical history - Touchscreen - UK Biobank Assessment Centre：  Vascular/heart problems diagnosed by doctor (6150) | 4 |
|  | Hypertension;  Essential hypertension | Medical conditions - Verbal interview - UK Biobank Assessment Centre：  Non-cancer illness code, self-reported (20002) | 1065, 1072 |
| Diabetes | Diabetes mellitus;  Insulin-dependent diabetes mellitus;  Non-insulin-dependent diabetes mellitus;  Malnutrition-related diabetes mellitus;  Other specified diabetes mellitus;  Unspecified diabetes mellitus | Summary Diagnoses - Hospital inpatient - Health-related outcomes:  Diagnoses - ICD10 (41270),  Date of first in-patient diagnosis - ICD10 (41280),  Diagnoses - ICD9 (41271),  Date of first in-patient diagnosis - ICD9 (41281) | ICD10: E10-E14, E10%-E14%;  ICD9: 250, 250% |
|  |  | Medical conditions - Health and medical history - Touchscreen - UK Biobank Assessment Centre：  Diabetes diagnosed by doctor (2443) | 1 |
|  | Diabetes;  Type 1 diabetes;  Type 2 diabetes | Medical conditions - Verbal interview - UK Biobank Assessment Centre：  Non-cancer illness code, self-reported (20002) | 1220, 1222, 1223 |
| Pelvic inflammatory disease | Inflammatory diseases of female pelvic organs;  Inflam. dis. - ovary, fallopian tube, pelvic cellular tiss., peritoneum;  Inflammatory diseases of uterus, except cervix;  Inflammatory disease of cervix, vagina and vulva | Summary Diagnoses - Hospital inpatient - Health-related outcomes:  Diagnoses - ICD10 (41270),  Date of first in-patient diagnosis - ICD10 (41280),  Diagnoses - ICD9 (41271),  Date of first in-patient diagnosis - ICD9 (41281) | ICD10: N70-N77, N70%-N77%;  ICD9: 614-616, 614%-616% |
|  | Pelvic inflammatory disease / pid | Medical conditions - Verbal interview - UK Biobank Assessment Centre：  Non-cancer illness code, self-reported (20002) | 1557 |
| HPV infection |  | Infectious Diseases - Blood assays - Assay results - Biological samples:  HPV 16 Definition I seropositivity for Human Papillomavirus type-16 (23068),  HPV 16 Definition II seropositivity for Human Papillomavirus type-16 (23075),  HPV 18 seropositivity for Human Papillomavirus type-18 (23069) | 1 |
| HIV disease | Human immunodeficiency virus [HIV] disease | Summary Diagnoses - Hospital inpatient - Health-related outcomes:  Diagnoses - ICD10 (41270),  Date of first in-patient diagnosis - ICD10 (41280) | ICD10: B20-B24, B20%-B24% |
|  | Hiv/aids | Medical conditions - Verbal interview - UK Biobank Assessment Centre：  Non-cancer illness code, self-reported (20002) | 1439 |
|  |  | Infectious Diseases - Blood assays - Assay results - Biological samples:  HIV-1 seropositivity for Human Immunodeficiency Virus (23064) | 1 |
| Oral contraceptive | Oral contraceptive | Female-specific factors - Sex-specific factors - Touchscreen - UK Biobank Assessment Centre:  Ever taken oral contraceptive pill (2784) | Yes: 1;  No: 0；  Missing: -1, -3, NA |
| HRT | Use of hormone-replacement therapy (HRT) | Female-specific factors - Sex-specific factors - Touchscreen - UK Biobank Assessment Centre:  Ever used hormone-replacement therapy (HRT) (2814) | Yes: 1;  No: 0；  Missing: -1, -3, NA |
| Tamoxifen | Tamoxifen | Medications - Verbal interview - UK Biobank Assessment Centre:  Treatment/medication code (20003) | 1140870164 |

/: quantitative data with no coding; ICD10: International Classification of Diseases, 10th Revision; ICD9: International Classification of Diseases, 9th revision; %: represent any number of characters; HRT: hormone-replacement therapy; HPV: human papillomavirus; HIV, Human immunodeficiency virus.

**SUPPLEMENTAL TABLE 6**

**Baseline characteristics of** **premenopausal females by use of statins**

| **Characteristics** | **Non-users**  **(n=** **52,849)** | **Statin users (n=** **1510)** | ***P* value** |
| --- | --- | --- | --- |
| **Age, years, median (IQR)** | 46 (43-49) | 48 (45-52) | <0.001 |
| **Race** |  |  | <0.001 |
| White | 48,474 (91.7) | 1307 (86.6) |  |
| Others | 4243 (8.0) | 200 (13.2) |  |
| Missing | 132 (0.2) | ﹤5 (0.2) |  |
| **Townsend deprivation index (quintiles)** | |  | <0.001 |
| 1 (least deprived) | 10,646 (20.1) | 209 (13.8) |  |
| 2 | 10,623 (20.1) | 231 (15.3) |  |
| 3 | 10,585 (20.0) | 270 (17.9) |  |
| 4 | 10,526 (19.9) | 328 (21.7) |  |
| 5 (most deprived) | 10,382 (19.6) | 472 (31.3) |  |
| Missing | 87 (0.2) | ﹤5 ( 0.0) |  |
| **Smoking status** |  |  | 0.117 |
| Never | 34,598 (65.5) | 971 (64.3) |  |
| Past | 13,050 (24.7) | 362 (24.0) |  |
| Current | 5091 (9.6) | 173 (11.5) |  |
| Missing | 110 (0.2) | ﹤5 (0.3) |  |
| **Alcohol use** |  |  | <0.001 |
| Daily or almost daily | 7223 (13.7) | 163 (10.8) |  |
| Three or four times a week | 12,238 (23.2) | 199 (13.2) |  |
| Once or twice a week | 15,016 (28.4) | 350 (23.2) |  |
| One to three times a month | 7624 (14.4) | 224 (14.8) |  |
| Special occasions only | 6725 (12.7) | 341 (22.6) |  |
| Never | 3989 (7.5) | 228 (15.1) |  |
| Missing | 34 (0.1) | 5 (0.3) |  |
| **Vigorous physical activity** |  |  | <0.001 |
| Low | 8066 (15.3) | 329 (21.8) |  |
| Moderate | 18,616 (35.2) | 449 (29.7) |  |
| High | 16,822 (31.8) | 357 (23.6) |  |
| Missing | 9345 (17.7) | 375 (24.8) |  |
| **Number of childbirth , median (IQR)** | 2 (0-2) | 2 (0-2) | 0.290 |
| **Number of abortion, median (IQR)** | 0 (0-1) | 0 (0-1) | 0.123 |
| **Comorbidities** |  |  |  |
| Hyperlipidemia | 458 (0.9) | 940 (62.3) | <0.001 |
| Ischemic heart disease | 220 (0.4) | 163 (10.8) | <0.001 |
| Ischemic cerebrovascular disease | 159 (0.3) | 87 (5.8) | <0.001 |
| Hypertension | 5609 (10.6) | 805 (53.3) | <0.001 |
| Diabetes | 717 (1.4) | 499 (33.0) | <0.001 |
| Obesity | 10,171 (19.2) | 747 (49.5) | <0.001 |
| Pelvic inflammatory disease | 770 (1.5) | 30 (2.0) | 0.115 |
| **Oral contraceptive** |  |  | <0.001 |
| Yes | 46,794 (88.5) | 1210 (80.1) |  |
| No | 5909 (11.2) | 292 (19.3) |  |
| Missing | 146 (0.3) | 8 (0.5) |  |

IQR, interquartile range. Data are n (%) unless otherwise indicated.
